# Progress of Signaling Pathways, Stress Pathways and Epigenetics in the Pathogenesis of Skeletal Fluorosis

**DOI:** 10.3390/ijms222111932

**Published:** 2021-11-03

**Authors:** Lichun Qiao, Xuan Liu, Yujie He, Jiaheng Zhang, Hao Huang, Wenming Bian, Mumba Mulutula Chilufya, Yan Zhao, Jing Han

**Affiliations:** Key Laboratory of Environment and Genes Related to Diseases, School of Public Health, Health Science Center, Xi’an Jiaotong University, Xi’an 710049, China; qlc978402409@163.com (L.Q.); liuxuan5241@stu.xjtu.edu.cn (X.L.); heyujie0406@stu.xjtu.edu.cn (Y.H.); zjh001210@stu.xjtu.edu.cn (J.Z.); 2196120510@stu.xjtu.edu.cn (H.H.); 2193410388@stu.xjtu.edu.cn (W.B.); mulutula@stu.xjtu.edu.cn (M.M.C.); zy97133@stu.xjtu.edu.cn (Y.Z.)

**Keywords:** skeletal fluorosis, fluoride, endemic disease, signaling pathways, epigenetics, endoplasmic reticulum stress, oxidative stress

## Abstract

Fluorine is widely dispersed in nature and has multiple physiological functions. Although it is usually regarded as an essential trace element for humans, this view is not held universally. Moreover, chronic fluorosis, mainly characterized by skeletal fluorosis, can be induced by long-term excessive fluoride consumption. High concentrations of fluoride in the environment and drinking water are major causes, and patients with skeletal fluorosis mainly present with symptoms of osteosclerosis, osteochondrosis, osteoporosis, and degenerative changes in joint cartilage. Etiologies for skeletal fluorosis have been established, but the specific pathogenesis is inconclusive. Currently, active osteogenesis and accelerated bone turnover are considered critical processes in the progression of skeletal fluorosis. In recent years, researchers have conducted extensive studies in fields of signaling pathways (Wnt/β-catenin, Notch, PI3K/Akt/mTOR, Hedgehog, parathyroid hormone, and insulin signaling pathways), stress pathways (oxidative stress and endoplasmic reticulum stress pathways), epigenetics (DNA methylation and non-coding RNAs), and their inter-regulation involved in the pathogenesis of skeletal fluorosis. In this review, we summarised and analyzed relevant findings to provide a basis for comprehensive understandings of the pathogenesis of skeletal fluorosis and hopefully propose more effective prevention and therapeutic strategies.

## 1. Introduction

Fluorine is one of the most common halogens, which usually exists in the environment as compounds. Fluorine or fluoride enters the human body primarily through drinking water, food, and air. Once absorbed into the bloodstream, it is easily transported throughout the body in the form of ions. More than 90% of absorbed fluoride is distributed in bone tissue, with the total amount of fluorine in a normal human body being approximately 2.6 g [1]. Fluoride has a dual effect on human well-being. Trace amounts of fluoride are required for the normal growth and development of the human body [2], while high intakes of fluoride can cause damage to tissues, organs, and systems. Dental fluorosis and skeletal fluorosis are characteristic manifestations of fluorosis. Long-term consumption of highly fluoridated water [2] and brick tea [3,4], or contaminated air and food entering the body owing to the use of high-fluoride coal as fuel [5,6] can lead to fluorosis. Unfortunately, there is no definite and effective treatment for patients with skeletal fluorosis, which severely threatens human health. Despite a declining incidence rate in recent years, fluorosis, particularly skeletal fluorosis, remains a severe public health problem in more than 40 countries worldwide. It is, therefore, crucial to recognize the mechanisms of skeletal fluorosis progression.

Fluorine has a strong bone affinity and tends to accumulate in bone tissue leading to skeletal fluorosis. Excessive fluoride will cause a series of damages to osteoblasts, osteoclasts, cartilage tissue, and bone mineralization in humans [7]. Fluorine has a bidirectional characteristic in bone production and resorption: it can not only cause osteosclerosis by enhancing osteogenic activity but also lead to osteoporosis by promoting bone resorption [8]. To date, the pathogenesis of skeletal fluorosis has not been fully explored. Over the years, researchers have focused on the various cellular regulatory mechanisms by which fluorine affects the bone turnover process [9]. Active osteogenesis and accelerated bone turnover have been proved to be crucial processes in the progression of skeletal fluorosis and the pathological basis for the diversity of osteogenic lesions [9,10]. Fluorine can induce differentiation and apoptosis of osteoblasts and osteoclasts, mainly by disrupting the dynamic balance of bone turnover, leading to skeletal damage and ultimately osteosclerosis, osteochondrosis, periosteal soft tissue ossification, osteoporosis, and degenerative changes in joints and cartilage in patients with skeletal fluorosis.

Researchers have made significant progress in understanding the regulation of signaling pathways, oxidative stress, endoplasmic reticulum stress, DNA methylation, and non-coding RNAs in recent years. By analyzing several studies on the relevant signaling pathways, stress pathways, epigenetics, and interaction networks among them involved in the pathogenesis of skeletal fluorosis, this review seeks to deepen understanding of the pathogenesis of fluorosis and hopefully propose more targeted prevention and therapeutic strategies.

## 2. Mechanism of Signaling Pathways in Skeletal Fluorosis

### 2.1. Effect of Wnt/β-Catenin Signaling Pathway on Skeletal Fluorosis

Wnt is a cytokine involved in various biological processes [11], and the canonical Wnt/β-catenin signaling pathway plays a crucial role in regulating osteoblast differentiation, osteogenic matrix formation, and bone homeostasis [12,13,14,15,16]. In the absence of Wnt ligands, however, the potential transcriptional mechanisms of the pathway cannot be activated [17]. The β-catenin inhibiting action of glycogen synthase kinase-3β (GSK-3β) is hindered when Wnt ligands (Wnt1, Wnt2, Wnt3a, and Wnt10a) act on the cell surface receptors Frizzled and low-density lipoprotein receptor-related protein 5/6 (LRP5/6), leading to decreased phosphorylation and degradation of β-catenin. β-catenin is thereby able to accumulate to a high enough level to enter the nucleus and interact with T-cell factor/lymphatic enhancer factor (TCF/LEF), thus regulating the expression of the target gene [17].

**Figure 1:**
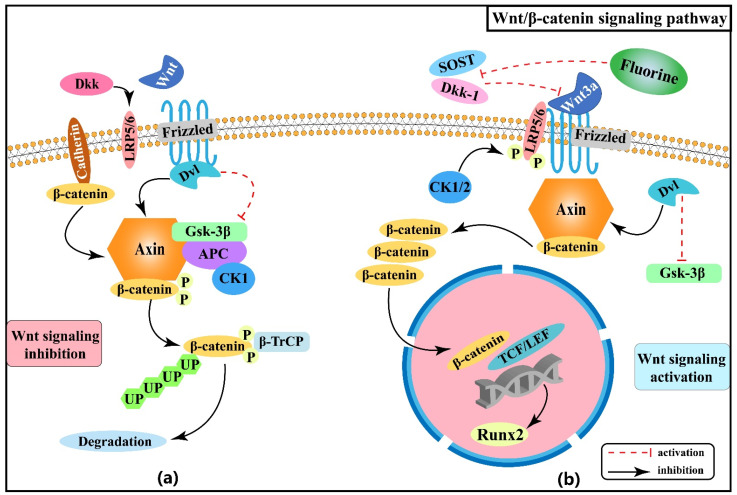
Wnt/β-catenin signaling pathway in the pathogenesis of skeletal fluorosis. (**a**) Wnt signaling is inhibited; (**b**) Wnt signaling is activated. GSK-3β: glycogen synthase kinase-3β; Dkk-1: dickkopf-related protein 1; LRP5/6: low-density lipoprotein receptor-related protein 5/6; TCF/LEF: T-cell factor/lymphatic enhancer factor; Runx2: Runt-related transcription factor 2; SOST: serum sclerostin.

Sun et al. confirmed that fluoride significantly increased osteoblast activity, as evidenced by the observed enhanced osteogenesis in their study [18]. Fluoride increased the expression of Wnt3a and β-catenin in rat osteoblasts; in addition, serum bone alkaline phosphatase (BALP) levels tended to increase with increasing doses of fluoride staining [19], suggesting that fluoride may be involved in the bone formation process by stimulating Wnt3a to up-regulate BALP expression. A recent study also found that fluoride can induce abnormal activation of the Wnt/β-catenin signaling pathway, causing the increased formation of cancerous bone in mice and protein expression of Wnt3a and phospho-Gsk-3β, as well as its downstream target gene Runt-related transcription factor 2 (Runx2). However, it must be noted that inhibition of β-catenin can suppress fluoride-induced Runx2 protein expression and resulting osteopathology [15], which suggests that β-catenin may be a key molecule in fluoride-induced aberrant osteogenesis. Serum sclerosing protein (SOST) and dickkopf-related protein 1 (Dkk-1) are inhibitors of Wnt/β-catenin signaling, and Wang [20], Liu [21], and Zeng et al. [22] have demonstrated that long-term or high-concentration exposure to fluoride can reduce SOST and Dkk-1 concentration, thereby inducing the activation of Wnt/β-catenin signaling, and finally leading to the progression and differentiation of osteoblasts. Figure 1 shows the association between Wnt/β-catenin signaling pathway and the pathogenesis of skeletal fluorosis.

Here, we review the mechanism of action of the Wnt/β-catenin signaling pathway in skeletal fluorosis, and it is evident that this signaling mediates the enhanced osteogenic effects of fluoride. Moreover, Wnt/β-catenin signaling pathway is connected to many signaling pathways in the control of osteoblast and chondrocyte proliferation and differentiation, such as PIK/Akt and hedgehog (Hh) signaling, and subsequent studies to explore their interactive regulation are even more valuable for comprehension of the pathogenesis of skeletal fluorosis.

### 2.2. Effect of Notch Signaling Pathway on Skeletal Fluorosis

Notch signaling pathway primarily mediates intercellular interactions and plays an integral part in determining cell fate and function together with the regulation of skeletal homeostasis [23]. The interactions of Notch receptors (Notch-1, 2, 3, and 4) with ligands (Jagged-1, Jagged-2, and Delta-like-1, 3, and 4) lead to a series of proteolytic cleavage and release of the Notch intracellular structural domain (NICD) into the cytoplasm [24,25]; NICD translocates to the nucleus and forms a complex with Epstein-Barr virus latency C promoter binding factor 1 (CBF-1)/repressor of hairless/Lag1 (CSL), which is known as recombination signal binding protein-Jκ (RBP-J) in mice, and Mastermind-like (Maml) to activate transcription of target genes (Hes-1, Hes-5, Hey-2, and Hey L) [26,27,28]. Notch signaling pathway regulates the development of osteoblast and osteoclast lineages and thus has a significant impact on skeletal development [29].

**Figure 2:**
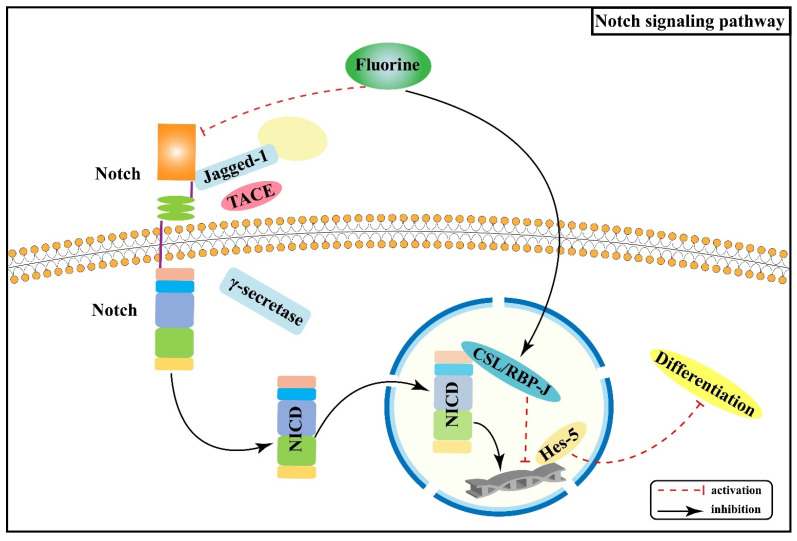
Notch signaling pathway in the pathogenesis of skeletal fluorosis. NICD: Notch intracellular structural domain; RBP-J: Recombination signal binding protein-Jκ; CSL: Epstein-Barr virus latency C promoter binding factor 1/repressor of hairless/Lag1.

Notch signaling can inhibit osteoblast differentiation [30,31], thereby maintaining bone marrow mesenchymal progenitors [27]. An animal experiment found that excessive fluoride exposure decreased the protein as well as mRNA expression levels of Notch-3 and Jagged-1 in rats, especially in osteoblasts [32], suggesting that fluoride can inhibit the Notch signaling pathway, thus promoting osteoblasts proliferation and differentiation, disturbed dynamic homeostasis of bone tissue, and the pathological manifestation of osteosclerosis. Another study [33] showed that RBP-J protein expression was increased and Hes-5 protein expression was decreased in bone tissue of rats dyed with fluoride, indicating that fluoride may promote osteoblast differentiation by promoting the expression of the transcriptional repressor RBP-J and enhancing the inhibitory effect on the downstream target gene Hes-5. Prior studies have suggested that the Notch signaling pathway mediates the enhanced osteogenic effects of fluorosis. Figure 2 shows the association between Wnt/Notch signaling pathway and the pathogenesis of skeletal fluorosis.

Notch signaling pathway displays a significant role in cell proliferation, differentiation, and apoptosis. Nonetheless, there are relatively few investigations on the relationship between Notch signaling pathway and skeletal fluorosis, mainly focusing on the mechanisms that regulate the proliferation and differentiation of osteoblasts. An in-depth investigation of the role of Notch signaling pathway in regulating other bone tissues and cells and its synergistic or antagonistic effects with other signaling pathways is of great significance for the pathogenesis, prevention, and treatment of skeletal fluorosis.

### 2.3. Effect of PI3K/Akt/mTOR Signaling Pathway on Skeletal Fluorosis

**Figure 3:**
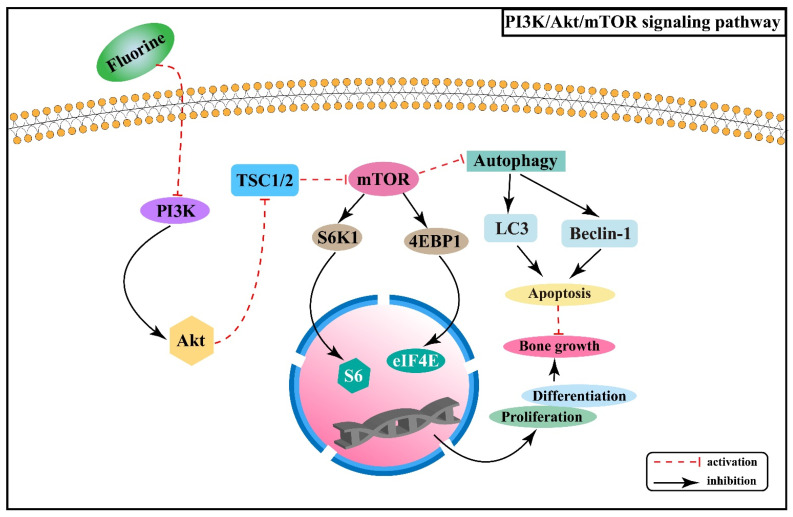
PI3K/Akt/mTOR signaling pathway in the pathogenesis of skeletal fluorosis. S6K1: ribosomal protein S6 kinase β-1; 4EBP1: 4E binding protein 1; LC3: light chain 3; TSC1/2: tuberous sclerosis protein complex1/2.

mTOR is a serine/threonine-protein kinase that can regulate a variety of biological processes. PI3K/Akt/mTOR signaling pathway is involved in the proliferation and differentiation of osteoblasts, osteoclasts, and chondrocytes [34,35,36]. Experiments in rats with chronic fluorosis have shown that the PI3K/Akt signaling may cause excessive proliferation and differentiation of osteoblasts and accelerate bone turnover, resulting in osteosclerotic skeletal fluorosis [37]. It was found that high-dose fluorine can inhibit the expression of mTOR protein in cartilage tissue, promote the expression of autophagy signature proteins, such as Beclin-1 and cytoplasm-associated protein light chain 3 (LC3), and enhance the apoptosis involved in the process of fluorine-induced cartilage damage [38]. After mTOR is activated, it phosphorylates the downstream target proteins ribosomal protein S6 kinase β-1 (S6K1) and eukaryotic translation initiation factor 4E binding protein 1 (4EBP1) to promote gene transcription and protein translation [39]. Fluorine can down-regulate PI3K/Akt/mTOR signaling and inhibit the phosphorylation of its downstream factors S6K1 and 4EBP1, thus promoting chondrocyte autophagy and inhibiting chondrocyte proliferation and differentiation [40]. The association between PI3K/Akt/mTOR signaling pathway and the pathogenesis of skeletal fluorosis is shown in Figure 3.

Skeletal fluorosis is characterized by a disrupted dynamic balance between bone resorption and formation and is intimately linked to regulating PI3K/Akt/mTOR signaling pathway, with a critical role in osteoblast proliferation and differentiation as well as chondrocyte autophagy. Understanding more about the changes of PI3K/Akt/mTOR signaling pathway and related signaling molecules in skeletal fluorosis, as well as its interaction with other signaling pathways, will promote a profound understanding of the pathogenesis of endemic fluorosis and provide a basis for the prevention and treatment of skeletal fluorosis.

### 2.4. Effect of Hedgehog Signaling Pathway on Skeletal Fluorosis

Hh signaling pathway consists of Hh protein, protein receptors Ptched (Ptch) and Smoothened (Smo), and the 5-zinc finger transcription factor Glis (Gli1, Gli2, and Gli3) [41]. There are three Hh homolog genes in mammals: Sonic Hedgehog (Shh), Indian Hedgehog (Ihh), and Desert Hedgehog (Dhh), which encode Shh, Ihh, and Dhh proteins, respectively. In the absence of Hh ligands, Ptch inhibits the activity of Smo, which further inhibits the expression of downstream genes in the nucleus [42]. When the Hh ligand binds to Ptch, its inhibitory effect on Smo is released, and the expression of target genes increases [42]. Research results have shown that removing Smo decreased the proliferation rate of chondrocytes, while upregulation could increase the rate [43].

**Figure 4:**
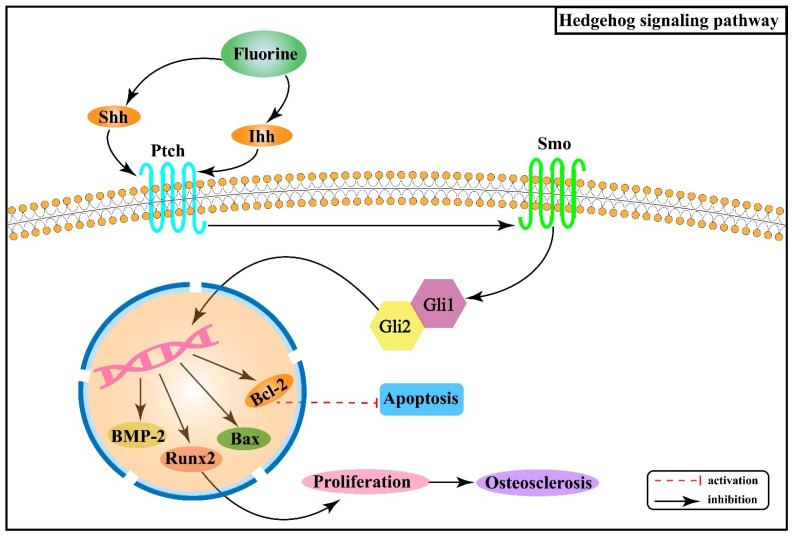
Hedgehog signaling pathway in the pathogenesis of skeletal fluorosis. Ptch: Ptched; Smo: Smoothened; Ihh: Indian Hedgehog; Shh: Sonic Hedgehog; Gli1 and Gli2: 5-zinc finger transcription factor Glis; Bcl-2: B cell lymphoma /leukemia-2; BMP-2: bone morphogenetic protein-2; Bax: B cell lymphoma/leukemia gene associated x.

Studies have revealed that fluoride stimulation increases the expression of Ihh in rat osteoblasts, and this signal, mediated by Smo, induces the expression of Runx2 after activating Gli2, which in turn promotes osteoblast proliferation and contributes to osteosclerosis [44]. Moreover, fluorine stimulation can cause chondrocytes to produce oxygen radicals, thus causing abnormal differentiation [45,46]. Shh is stimulated and released when oxidative stress occurs, synergizing with oxidative stress to promote the B cell lymphoma/leukemia-2 (Bcl-2) anti-apoptotic protein [47]. Animal experiments demonstrated that with the expression of Shh, Smo, bone morphogenetic protein-2 (BMP-2) and B cell lymphoma/leukemia gene associated × (Bax) protein gradually increased in rat cartilage tissues with increasing fluoride staining concentration, while Bcl-2 protein expression gradually decreased [48]. Figure 4 shows the association between Hh signaling pathway and the pathogenesis of skeletal fluorosis.

The above studies suggest that the Hh signaling pathway is closely associated with oxidative stress and apoptosis and regulates chondrocyte ossification and apoptosis in skeletal fluorosis. In addition, the available data reveal that Hh signaling is also related to Wnt/β-catenin, Notch, Bmp, and other signaling pathways, and its specific regulatory mechanisms in the pathogenesis of skeletal fluorosis that damages cartilage tissue remain to be investigated in depth.

### 2.5. Effect of Hormones and Their Receptor Signaling Pathways on Skeletal Fluorosis

**Figure 5:**
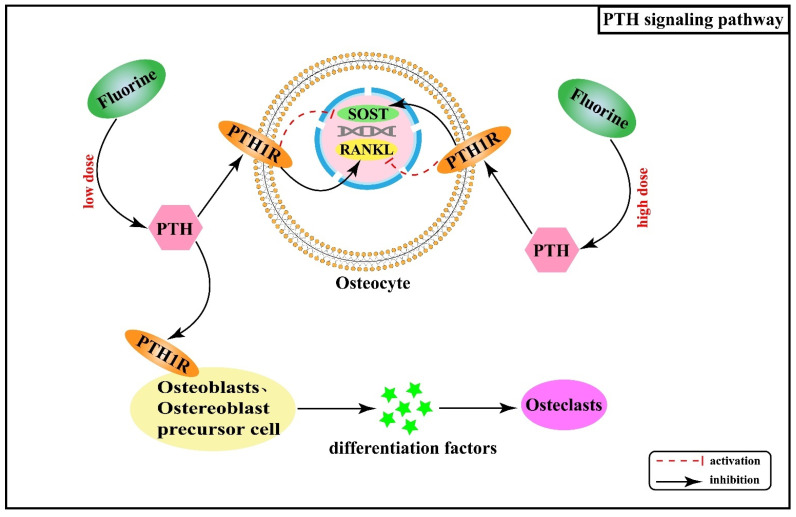
PTH signaling pathway in the pathogenesis of skeletal fluorosis. PTH: parathyroid hormone; PTH1R: parathyroid hormone receptor; RANKL: receptor activator of nuclear factor-κB ligand.

Over the years, systematic studies of the mechanisms of fluorosis in animals and humans have revealed that many calcium-regulating hormones and cytokines are also involved in mediating abnormal osteogenic and osteolytic bone metabolism in skeletal fluorosis, such as parathyroid hormone (PTH) and transforming growth factor β (TGF-β). PTH is a polypeptide hormone responsible for regulating calcium homeostasis in the body and exerts an active role in regulating bone turnover. Its effects on cells are mediated by the G protein-coupled receptor (PTH receptor, PTH1R) expressed by target cells, including osteoblasts, osteoblast precursor cells, and osteocytes, but excluding osteoclasts [49,50]. Therefore, PTH acts indirectly on osteoclasts by binding to PTH1R on the surface of the osteoblast cell line and secreting osteoclast differentiation factors. Additionally, a study found that serum PTH levels gradually increased with increasing fluoride exposure [51]. PTH can regulate fluoride action through its effects on the expression of SOST and receptor activator of nuclear factor-κB ligand (RANKL) in osteocytes. By up-regulating RANKL and inhibiting SOST, PTH enhances the effectiveness of low-dose of fluoride in bone turnover promotion while having opposite effects on SOST and RANKL in the cases of high fluoride doses [52]. Figure 5 shows the association between PTH signaling pathway and the pathogenesis of skeletal fluorosis.

In addition, PTH, as one of the important hormones regulating calcium and phosphorus metabolism in the body, exerts a bidirectional regulatory effect on bone metabolism. High doses of PTH promote bone resorption, while at low doses, it stimulates bone formation. Furthermore, animal and clinical studies have revealed that when PTH is given intermittently subcutaneously, it stimulates bone formation, increases bone density in long bones and vertebrae, and improves the bone quality [53,54]. It follows that the use of PTH in the treatment of osteoporosis has been approved by the US Food and Drug Administration and other organizations [54].

Insulin is a multifunctional protein hormone that acts by binding to the insulin receptor (IR) in most tissues. IR and intracellular signaling pathways are the main components of the insulin signaling pathway [55]. Fulzele et al. [56] found that IR exists in osteoblasts, and insulin promotes bone formation by inhibiting Twist2 (Runx2 inhibitor) after interacting with cell surface receptors. Studies have pointed out that fluoride can stimulate IR expression and osteoblast function in vitro and affect insulin secretion, activity, and sensitivity [57,58]. Furthermore, insulin state in turn interferes with bone formation and absorption [57,58]. Another study showed that insulin plays a vital role by mediating IR signals, and insulin-like growth factor-1 (IGF-1) may play a role in bone turnover induced by excessive fluoride by regulating IR or its downstream [59]. All these studies suggest that insulin exerts an essential role in fluoride-induced bone pathogenesis.

### 2.6. Interactive Regulatory Networks among Signaling Pathways Involved in Skeletal Fluorosis

**Figure 6:**
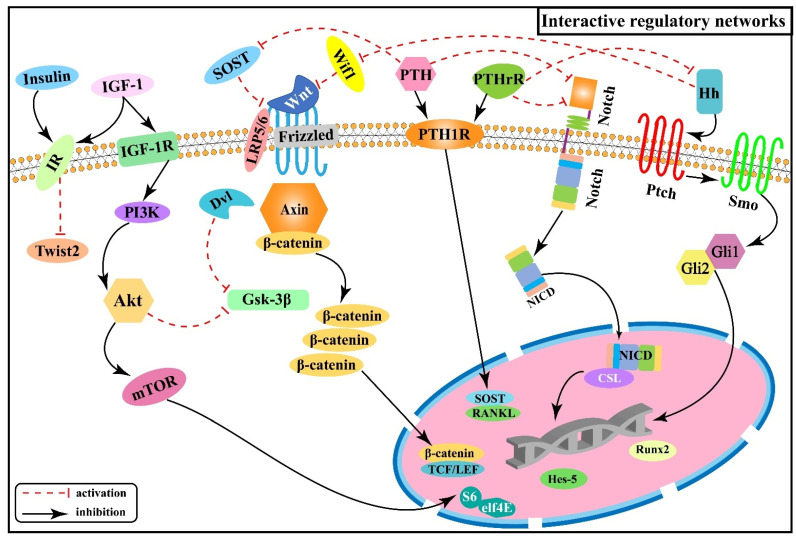
Interactive regulatory networks among Wnt/β-catenin, Notch, PI3K/Akt/mTOR, Hh, PTH, and insulin signaling pathways involved in skeletal fluorosis.

In the pathogenetic progression of skeletal fluorosis, both local signaling pathways and hormones are involved. Activation or inhibition of signaling is not accomplished linearly within the single signaling pathway mentioned above alone, but rather the signaling pathways interact to form an interactive regulatory network that acts to control biological processes in a synergistic or antagonistic manner [60]. Figure 6 illustrates the interactive regulatory networks formed among Wnt/β-catenin, Notch, PI3K/Akt/mTOR, Hh, PTH, and insulin signaling pathways involved in skeletal fluorosis.

PTH can bind to the same receptor (PTH1P) as the parathyroid hormone-related peptide (PTHrP) synthesized by osteoblasts and mediates bone formation and accelerated bone turnover through multiple pathways. And SOST expressed by osteoblasts is a key negative regulator of bone formation [61,62] and acts as an upstream inhibitor of the Wnt/β-catenin signaling pathway by binding to Wnt and blocking its interaction with the cell surface receptors Frizzled or LRP5/6 to inhibit the activation of β-catenin downstream in the Wnt signaling pathway. Whereas PTH causes transcriptional suppression of the osteocyte marker gene SOST and downregulates SOST protein expression [63], thereby activating the Wnt/β-catenin signaling pathway. Experiments revealed that the expression of Ihh in rat growth plate chondrocytes decreased with increasing fluorine concentration, while the expression of PTHrP showed an increasing trend, suggesting that fluorine reduces the expression of Ihh by up-regulating PTHrP, inhibiting the Ihh/PTHrP negative feedback loop to affect chondrocyte proliferation and differentiation, and inhibiting the normal process of osteogenesis within cartilage [64]. In addition, it was shown that PTH can down-regulate the expression of the Notch signaling pathway, and this inhibitory effect may be achieved by down-regulating intracellular cAMP/PKA signaling, reducing the expression of receptor Notch-1 and ligand Jagged-1 of the Notch signaling pathway, which in turn affects the expression of Runx2 [65]. The researchers found positive regulation among PTHrP, NICD, and Jagged-1 proteins after transfecting epiphyseal stem cells with PTHrP over-expression and lentiviral interference vectors, suggesting that PTHrP may act through influencing the Notch signaling pathway [66]. The above information indicates that the Wnt/β-catenin, Hh, and Notch signaling pathways are associated with the PTH signaling pathway and act downstream of the PTH signaling.

Both Wnt/β-catenin and Ihh signaling pathways control the proliferation and differentiation of osteoblasts and chondrocytes at multiple stages [12,13,14,15,44,45,46]. One study tested the genetic relationship between Wnt and Hh signaling by generating double mutant mice with results revealing that Wnt/β-catenin signaling acts downstream of Hh signaling in enhancing bone formation [67], in agreement with another study [68]. Subsequently, the expression of genes associated with different stages of osteoblast differentiation was also examined, showing that β-catenin is required downstream of Ihh in promoting osteoblast maturation [67]. It has been demonstrated that Wif1 (Wnt inhibitory factor 1) exerts biological effects by mediating and regulating Shh/Wnt/β-catenin signaling [69], as well as Wif1 can effectively block the activation of the canonical Wnt signaling pathway in chondrocytes by binding to Wnt ligands (Wnt3a, etc.) [69,70,71], speculating Hh signaling may exert inhibitory effects on downstream Wnt/β-catenin signaling through Wif1 in the pathogenesis of skeletal fluorosis. At the same time, the Wnt/β-catenin signaling is also interactively regulated with the PI3K/Akt signaling. It was shown that PI3K/Akt negatively regulates Gsk-3β activity, thereby inhibiting the phosphorylation of β-catenin [72], which suggests a role for Wnt/β-catenin signaling acting downstream of PI3K/Akt. To determine this role, the researchers further analyzed the expression of Wnt-regulated genes, such as Dkk1 and Sfrp1 (secreted frizzled-related protein 1), showing that PI3K signaling activates these genes by peptide-mediated α5β1 integrin priming in mesenchymal skeletal cells [73]. Moreover, PI3K/Akt signaling is also regulated by insulin-related signaling. IGF-1 is a principal growth-promoting signal for vertebrate skeletal development [74] and as a specific ligand can activate the PI3K/Akt signaling pathway by binding and phosphorylating the membrane IGF-1 receptor (IGF-1R), leading to osteoblast differentiation and proliferation [75].

At present, studies on the relevant signaling pathways involved in skeletal fluorosis are still predominantly single lineage studies. Nevertheless, active osteogenesis and accelerated bone turnover as crucial processes in the progression of skeletal fluorosis are regulated by sophisticated networks of multiple signaling pathways. The profound exploration of the interactive regulatory mechanisms among signaling pathways in skeletal fluorosis will facilitate the development of specific targeted therapeutic measures.

## 3. Mechanism of Stress Pathways in Skeletal Fluorosis

### 3.1. Effect of Endoplasmic Reticulum Stress on Skeletal Fluorosis

The endoplasmic reticulum (ER) is essential for protein synthesis and secretion in eukaryotic cells [76]. When cells are subjected to certain external stimuli, the ER generates a series of regulatory mechanisms, generating endoplasmic reticulum stress (ERS) [77]. At the same time, ERS can protect cells from damage by stimulating the unfolded protein response (UPR) [78] or initiating the apoptotic program to ensure the survival of the organism [79,80]. Proteomic studies have revealed that the expression of immunoglobulin heavy chain binding protein (BiP), also known as glucose-regulated protein 78 (GRP78), protein disulfide isomerase (PDI), proteasome 26S ATPase, and thioredoxin (Trx) is up-regulated in fluoride-stained osteoblasts, and these proteins play key roles in protein folding of ER [81]. The results provide clues that ERS and UPR may be involved in the pathogenesis of skeletal fluorosis. URP is mediated by Bip and three response sensing proteins, including protein kinase-like ER kinase (PERK), inositol-requiring enzyme 1 (IRE1), and activating transcription factor 6 (ATF6) [82].

PERK is a serine/threonine-protein kinase that can be activated by accumulating misfolded or unfolded proteins on the ER [83]. It can reduce the newly synthesized proteins of the ER by activating eukaryotic initiation factor 2a (eIF2a) and activating transcription factor 4 (ATF4) pathways, thereby reducing ER load, and even inducing C/EBP homologous protein (CHOP) protein synthesis to stimulate the apoptotic program [84,85,86,87]. In addition, PERK can also directly activate nuclear factor erythroid 2-related factor 2 (Nrf2) to antagonize apoptosis to protect cell survival [88]. A study has shown that fluoride exposure activates the PERK signaling pathway, leading to activation of ATF4 and Nrf2 and up-regulating the expression of genes related to bone turnover in osteoblasts [89]. Meanwhile, the study also showed that ATF6 and IRE1 signaling factors mediated a less pronounced effect of ERS, suggesting that fluoride exposure mediates osteoblast damage mainly through the PERK signaling pathway [89].

To further clarify the role of the PERK signaling pathway, changes in osteogenic and osteolytic gene expression were studied in osteoblastic cell lines before and after PERK gene interference, and the results showed that fluoride stimulated the protein expression of PERK, Nrf2, osteoprotegerin (OPG), and Runx2 in PERK siRNA-transfected cells to a certain extent [90]. The above findings suggest the vital role of the PERK/Nrf2 pathway in the activation mechanism of fluorine-induced osteoblast and osteoclast. Nevertheless, studies on the IRE1 pathway and ATF6 pathway concerning skeletal fluorosis are still scarce and need to be further explored to provide new ideas and theoretical basis for preventing and treating skeletal fluorosis.

### 3.2. Effect of Oxidative Stress on Skeletal Fluorosis

Oxidative stress is considered to be an essential mechanism in the pathogenesis of fluorosis. Intake of large amounts of fluoride can cause an imbalance between antioxidant defense mechanisms and free radical levels, leaving cells in a state of oxidative stress [91]. In fluorosis, oxidative stress increases reactive oxygen species (ROS) and is accompanied by reduced antioxidant enzyme activity and increased lipid peroxides [92,93]. Nevertheless, excessive ROS can disrupt the dynamic balance between bone formation of osteoblasts and bone resorption of osteoclasts, leading to the development of skeletal fluorosis [94]. A study has shown that osteoblasts treated with low fluorine concentrations are in a low-level oxidative stress state and maintain redox homeostasis by activating the nuclear factor E2-related factor 2-antioxidant responsive element (Nrf2-ARE) signaling pathway, which prevents cells from apoptosis or death [95]. In contrast, when treated with high concentrations (4.00 mmol/L) of fluoride, the intracellular antioxidant system and signaling pathways were disrupted or disintegrated, leaving the cells in a decompensated state, and undergoing severe oxidative damage, even inducing apoptosis in osteoblasts [95]. Another study noted that intracellular ROS levels increased significantly after sodium fluoride treatment, and osteoblasts presented apoptotic morphological changes such as chromatin condensation and DNA fragmentation [96].

In addition, fluoride can likewise participate in the regulation of osteoclast proliferation through the oxidative stress pathway. Calcineurin (CaN) is a serine/threonine phosphatase dependent on calcium (Ca) and calmodulin (CaM), which is involved in the regulation of osteoclast differentiation and proliferation [97]. Animal experiments have demonstrated that excessive fluoride exposure led to increased CaN mRNA and protein expression levels and serum CaN activity in rat bone tissue, but its upstream regulators Ca and CaM had no significant differences between the control and fluoride-infected groups [98,99]. However, malondialdehyde (MDA) levels of the fluoride-infected group were significantly higher than the control group, and oxidative stress levels were also positively correlated with CaN activity [99,100], suggesting excess fluoride may stimulate elevated CaN activity in the organism through the oxidative stress pathway, further leading to increased osteoclast production in rat bone tissue.

The above studies have illustrated that fluoride can induce osteoblast apoptosis and osteoclast proliferation through the oxidative stress pathway, providing evidence for the pathogenesis of skeletal fluorosis. However, the molecular mechanisms of fluoride effects on osteoclasts are still relatively poorly investigated, and how fluoride affects osteoclast proliferation and differentiation through the oxidative stress pathway has not been comprehensively clarified, and further in-depth studies are necessary. Besides, since both endoplasmic reticulum stress and oxidative stress are associated with the pathogenesis of skeletal fluorosis, further studies are needed to explore the interrelationship between them to provide new insights into the pathogenesis of skeletal fluorosis.

## 4. Mechanism of Epigenetics in Skeletal Fluorosis

### 4.1. Effect of DNA Methylation on Skeletal Fluorosis

DNA methylation is one of the earliest and most common epigenetic modifications, and it plays an important role in the pathogenesis of skeletal fluorosis. P16 protein is a key factor of cell cycle regulation in the G1/S phase, competitively inhibits the cell progression from G1 phase to S phase and blocks abnormal activation of osteoblasts [101,102]. In fluorosis model experiments, promoter hypermethylation of the p16 gene inhibited its mRNA transcription and protein expression, while decreased p16 protein expression reduced its G1/S phase blocking effect, potentially providing an important molecular mechanism for the altered proliferative capacity and cell cycle distribution of osteoblasts caused by fluorine [103]. In addition, research has revealed that fluoride causes promoter DNA hypermethylation of the BMP1, METAP2, MMP11, and BACH1 genes, then RNA expression levels are down-regulated, thereby promoting skeletal fluorosis [104].

An animal experiment has shown that estrogen receptor α (ERα) mRNA expression is enhanced when the promoter methylation level of the ERα gene is inhibited in osteoblasts, thereby promoting osteoblast proliferation and differentiation [105]. In addition, the promoter methylation level of the ERα gene is negatively correlated with urinary fluoride concentration, suggesting that the pathological bone changes induced by fluoride exposure in men were related to the promoter hypomethylation of the ERα gene [106]. In conclusion, due to the diversity of fluoride regulation of bone damage and cellular DNA methylation, further works on the epigenetics of cellular regulation-related genes in fluorosis are needed.

### 4.2. Effect of Non-Coding RNAs on Skeletal Fluorosis

Following extensive research on the molecular mechanisms of abnormal bone metabolism in skeletal fluorosis at the molecular biology level, the role of microRNAs (miRNAs) is gradually being discovered. miRNAs as non-coding RNAs are post-transcription regulators of gene expression. miRNAs negatively regulate the expression of their target mRNAs and can inhibit the expression of mRNAs [107]. Fluoride exposure in human osteosarcoma cells can affect the expression of genes related to bone metabolism through the miRNA pathway [108]. Cyclin D1 is a protein that regulates the cell cycle by facilitating the cell transition from G1 phase to S phase and accelerating cell proliferation [109]. Down-regulation of mir-486-3p can affect the expression of cyclin D1 and further regulate osteoblast proliferation and activation [110]. Studies have shown that fluoride exposure induces the down-regulation of miR-4755-5p and Let-7c-5p, promoting fluoride-induced osteoblast proliferation and activation by regulating cyclin D1 expression [111,112].

It was pointed out that miR-29a promotes the differentiation process of osteoblasts by targeting and inhibiting the expression of Dkk-1 [113]. The specific mechanism is that the canonical Wnt signaling pathway can induce transcription of miR-29a, which can enhance the Wnt signaling pathway by downregulating Dkk-1 (an antagonist of Wnt signaling), thereby regulating the osteoblast differentiation process [113,114]. Another study also showed that the expression level of miR-27 was positively correlated with that of β-catenin, and it could activate Wnt signaling through the accumulation of β-catenin protein, thereby promoting osteoblast differentiation [115]. This suggests that miR-27 could be a possible target for the development of drugs against skeletal fluorosis. In conclusion, the regulatory networks of non-coding RNAs in fluorosis are complex, and further investigations are needed to elucidate their regulatory mechanisms on specific target mRNAs. Meanwhile, exploring the interactions between miRNAs and other signaling pathways may provide new insights into the therapeutic strategies for skeletal fluorosis.

## 5. Conclusions

In summary, skeletal fluorosis is a chronic and progressive endemic disease with severe risks to human health. Exposure to a specific duration or dose of fluoride can alter osteoblasts, osteoclasts, and chondrocytes’ function, differentiation, and proliferation and lead to skeletal fluorosis by disrupting the balance between bone formation and resorption. Signaling pathways, stress pathways, epigenetics, and interactive regulatory mechanisms play crucial roles in the pathogenesis above. With the development of multi-level and multi-faceted studies in recent years, considerable progress has been made at the molecular biological level in understanding the pathogenesis of skeletal fluorosis. Unfortunately, although a great deal of research has been carried out, there are no clear and effective treatments for patients with skeletal fluorosis.

The treatment of skeletal fluorosis in traditional Chinese medicine (TCM) focuses on reducing fluoride concentrations in the body and improving autogenous regulatory mechanisms, while western medicine aims to reduce the toxicity of fluoride to the body and repair the damage through specific interventions [116]. Studies have suggested that chemical elements such as calcium and magnesium can antagonize fluoride toxicity by binding fluoride in the intestine to form low-solubility complexes for excretion, thereby reducing fluoride absorption [117]. Antioxidants have also been used to treat skeletal fluorosis, and concomitant intake of calcium, vitamin C, and vitamin D can antagonize fluoride toxicity [118]. Additionally, some components of Chinese herbal medicine have antioxidant effects and can also effectively antagonize the oxidative stress caused by fluorosis, thereby reducing the degree of fluorosis [94,119]. Considering the combination of TCM and Western medical treatment ideas for skeletal fluorosis may yield better effects. At the same time, it is more important to continue in-depth research on the complex pathogenesis of skeletal fluorosis and explore new targeted therapies at the molecular level to treat skeletal fluorosis.

Since there is no specific effective treatment to cure skeletal fluorosis completely, prevention and control of skeletal fluorosis by using safe drinking water and reducing the use of high fluoride coal burning is currently considered the ideal approach [2]. Particularly, clarifying the pathogenesis of skeletal fluorosis and exploring effective targets against fluorine toxicity are of great importance to scientifically prevent and control the occurrence, development, and prevalence of skeletal fluorosis and reduce the risk to human health.

## Data Availability

Not applicable.
